# Plate waste of adults in the United States measured in free-living conditions

**DOI:** 10.1371/journal.pone.0191813

**Published:** 2018-02-14

**Authors:** Brian E. Roe, John W. Apolzan, Danyi Qi, H. Raymond Allen, Corby K. Martin

**Affiliations:** 1 Department of Agricultural, Environmental and Development Economics, Ohio State University, Columbus, OH United States of America; 2 Pennington Biomedical Research Center, Baton Rouge, LA United States of America; University of Tennessee Health Science Center, UNITED STATES

## Abstract

We analyze food-item level data collected from 50 adults from the United States using the Remote Food Photography Method® to provide the first estimates of plate waste gathered from adults across multiple consecutive meals and days in free-living conditions, and during laboratory-based meals with fixed food items and quantities. We find average plate waste in free-living conditions is 5.6 grams (7.7 kcals) per item and that 3.3% of all food selected is returned as plate waste, where the percent waste figure is substantially lower than previously published plate waste estimates gathered primarily from dine-out settings in the United States such as buffets and institutional settings with limited-choice meals (e.g., school cafeterias). Plate waste from the same participants during the laboratory-based meals is significantly higher with an average of 203.2 grams of solid plate waste per meal (531.3 kcals) or 39.1% of the food provided, which is similar to the plate waste percentages found reported in some school cafeteria settings. The amount of plate waste generated in free-living conditions is significantly positively associated with portion size selected for an item. In a multivariate analysis that controls for macronutrient profile, items selected from the vegetables, fats/oils/dressings, and grains categories are associated with significantly greater amounts of plate waste per item. We find no significant associations between free-living plate waste and gender, age, race or body mass index but find that women leave more plate waste in the lab meal where portion sizes are pre-determined by the researcher and similar for all respondents. We discuss possible implications of these findings for programs focused on reducing plate waste and food waste among consumers.

## Introduction

Countries around the world have resolved to reduce food waste in an attempt to advance food security, environmental sustainability and economic efficiency goals [1–4]. More than 40% of food waste occurs at the retail and consumer level in industrialized economies [5]. Plate waste–food that is served on individual plates but not consumed–is among the largest sources of avoidable food waste generated within households and represents about 30% of all avoidable household food waste in the United Kingdom, the country for which the most detailed analysis of household food waste is currently available [6–7]. Hence, understanding the amount, composition and patterns of plate waste in household settings may provide key insights for addressing the larger issue of avoidable household food waste.

Most plate waste studies from the United States have been conducted among children in schools that participate in the National School Lunch Program (NSLP). Buzby and Guthrie [8] summarize literature from a number of studies published in 2000 or earlier, and estimate that 12% of calories served in NSLP meals are plate waste and discarded. More recent studies of student plate waste from NSLP meals [9–16] yield higher estimates of plate waste ranging from a low of 12% among 6th graders for sliced and shredded cheese [15] to 74% for the vegetable components of meals served to middle schoolers [13] with the majority of plate waste estimates from these more recent studies exceeding 20%. Other studies involving students in different settings yield lower estimates of plate waste, including an estimate of 11% among girls aged 9–13 across both school and non-school meals captured via a mobile food record [17] and an estimate among high school students at a school lunch buffet of 11% for meals served on standard plates and 20% for meals served on disposable plates [18]. Our group has measured food waste over consecutive days in several school cafeteria settings and has found that plate waste varied between 9% and 28% [19–20].

While each study provides key information on plate waste, estimates taken from students may not yield estimates that translate well to the broader food sector as (1) it only considers consumption patterns of students, (2) most studies involve meals with predetermined portion sizes (rather than self-selected by the participant) chosen from a limited set of available menu items and (3) many students do not bear the cost of the food that is chosen because school meals are free or paid for by parents. Studies featuring adults that report percent waste figures range from 7% to 18% [21–25] while studies that only report amounts range from 15g–124g per person per meal [18, 26–29].

The studies featuring participants 18 years and older occur either in a no-price fixed-menu buffet setting [18, 21], in fixed-price all-you-can-eat buffets [22–23], in buffets included as part of lodging costs [29], or pre-paid meal plan settings [24–28]. However, less than one-third of calories are consumed in food-away-from-home settings in the United States [30] and, among these eating occasions, buffet-style restaurants account for less than 3% of sales from that sector [31–32]. Further, across all plate waste studies, most consider only midday meals (exceptions include [17]–multiple meal times, [22]–evening meals, and [29]—breakfast). In the United Kingdom, studies document that more food is wasted during evening meals [7].

The research closest to our work features detailed household diary data collected and analyzed in the United Kingdom for all sources of food wasted by a household while dining at home [7]. For the 284 respondents that maintained diaries in 2007, a separate ‘left on plate’ category was estimated, which corresponds to our measure of plate waste. The figure for per person avoidable food waste per year in the United Kingdom is 83.8kg (the 66.2kg/year figure reported for 2012 was described as 21% less than the 2007 figure, which yields 66.2/(1–0.21) = 83.8, where both figures are published in [33] (pg. 13). Coupling this calculation with the assumption of 3 meals per day and the 2007 estimate of 30% avoidable food waste due to plate waste [7] yields an estimate of 23g per meal per person wasted. Quested and Parry [33] have indicated a general reduction in household food waste since 2007 though constructing a comparable plate waste measure for these later years is difficult as the estimate for the percent of all food waste as ‘left on plate’ has not been updated since 2007.

In this study we analyze food-item level data collected from 50 adults from the United States using the Remote Food Photography Method® (RFPM) to provide estimates of plate waste gathered from adults in free-living conditions across multiple meals and days. By free-living, we mean that respondents recorded plate waste data as part of their normal day-to-day routine featuring food selections chosen from foods acquired in their normal manner rather than in a meal setting designed by the researcher. Respondents had no direction from the researcher concerning the types or amounts of food selected nor was data collection limited to settings in which food selection was constrained by institutional demands, e.g., school cafeterias or limited-menu buffets, or restricted to only meals eaten at home, e.g., household food waste diaries. In addition, we have data recorded using the same method from two meals prepared and provided by the researchers to each respondent in a laboratory setting. Hence, we have the first known instance where plate waste is recorded for the same respondents both in free-living and lab conditions.

## Materials and methods

### Measurement

Data were collected from participants during all eating occasions over approximately one week in participants’ free-living conditions; this includes all times of day, weekends as well as weekdays, and food at home as well as food away from home. Data were also collected at two meals provided during laboratory-based sessions. The data were acquired via the RFPM®, which has been previously described and validated [34–35]. Briefly, participants used smartphones to capture images of their food selection before each eating episode and plate waste after each eating episode including any instances of multiple servings (e.g., second helpings) and any episodes involving beverages that contained calories. Data acquisition is monitored in near real-time. If respondents fail to send data over a certain length of time the researchers contact and prompt the respondent to fill in a paper diary entry that is later integrated into the data file. For this sample, we found that such back-up methods were used on about 10% of days and accounted for about 10% of the energy intake estimates. Importantly, we found that the data, which includes the back-up method data, were complete since we tested energy intake estimated with food photography to the doubly labeled water technique and found the error to be very small (less than 4%) [35]. If the identity of the food was not clear from the image, participants identified the food within the smartphone app to facilitate our linking of the food to a nutrient database, namely, the United States Department of Agriculture’s Food and Nutrient Database for Dietary Studies (USDA FNDDS version 3.0 [36] was in use when these data were collected). The first number of the FNDDS food code designates the food group of the food item. The USDA has nine food groups which include: (1) milk and milk products, (2) meat, poultry, fish and mixtures, (3) eggs, (4) legumes, nuts and seeds, (5) grain products, (6) fruits, (7) vegetables, (8) fats, oils, and salad dressings, and (9) sugars, sweets, and beverages.

Compared to alternative approaches such as written food diaries, participant burden is reduced as participants need only capture and send cell-phone photos of their plates before and after eating, and these procedures are now streamlined via the SmartIntake® app. The photos are automatically received by our server for analysis, where the research team uses a computer-assisted approach to identify a match for each food in a nutrient database and estimates portion size based on established and validated procedures [34, 35, 37, 38]. The analyst enters the portion size for food selection and plate waste, and the computer system calculates the energy and nutrient composition for food selection, plate waste, and food intake, where food intake is calculated as selection minus waste.

Participants were also asked to come to the research center two times to consume laboratory meals. These meals were weekday lunches where participants were asked to take a picture of their food selection and plate waste. Participants were instructed to eat normally prior to arrival; neither meal occurred during the free-living conditions data collection period. At enrollment, participants selected from a limited offering of sandwiches with different types of lunchmeat and condiments, and they selected a beverage. Thus, lunch meat sandwiches (ranging from 230 g to 315 g with 83% being modal at 315 g, and ranging from 499 kcal to 626 kcal with 36% being modal at 587 kcal)) and drinks (ranging from 0 g to 1040 g with 37% being modal at 208 g, and ranging from 0 kcal to 208 kcal with the 24% being modal at 7 kcal) varied based on the preferences of the participant. The meals also consisted of a fixed amount of pretzels (Rold Gold; 42.5 g, 161.5 kcal)), fruit cup (113 g, 64.4 kcal), and cookies (Famous Amos; 56 g, 272.7 kcal). 44 of the 50 respondents participated in both meals.

### Sampling

The study sample includes 50 adults age 18 to 65 years recruited from the Baton Rouge, Louisiana region. Participants were generally healthy and were not diagnosed with chronic health conditions or diseases, including diabetes, cardiovascular disease, and cancer. Participants were also free of medication use that affected body weight and were weight stable (≤500g weight change over approximately one week).

### Analysis

Results are analyzed in Stata (version 14.2). Demographic variables available include gender, age, race and Body Mass Index where summary statistics for these variables are listed in Table 1. The BMI measurement used in this analysis is calculated on the day prior to initiating RFPM® using height and weight captured in a clinical research unit on a calibrated stadiometer and scale.

**Table 1:** Summary statistics by participant (N = 50).

Characteristic		% or Mean ± S.E
Gender		
	Male (%)	12
	Female (%)	88
Age (years)	18–29 (%)	26
	30–49 (%)	38
	50+ (%)	36
	Mean	41.0 ± 1.8
Race	White (%)	62
	African American (%)	38
Days	# of days of data recorded	5.48 ± 0.16
Height (cm)	Male	178.0 ± 72.7
	Female	162.2 ± 24.4
Weight (kg)	Male	99.4 ± 40.6
	Female	82.0 ± 12.4
BMI (kg/m^2^)	Male	30.9 ± 12.6
	Female	31.2 ± 4.7

Food item variables include the number of servings selected, grams selected, caloric density (calories per serving), the FDNNS food group, and the percentage of energy from protein, fat, and carbohydrates. The definition of servings for each type of food is based upon the most appropriate portion size image that, in some instances, leads to a high serving count for a small amount of food (e.g., an individual baby carrot is counted as one serving). Thus servings are not necessarily manufacturer standard servings nor USDA standard servings, but are likely the standard that allows for the best portion size estimation (i.e. a smaller portion will likely have a smaller serving whereas a larger portion will likely have a larger serving). Summary statistics for food-level items are presented in Table 2 (n = 2,400 where 22 of the original 2,422 observations are omitted due undocumented quantities of the food selected). Pairwise group differences are tested with a *t*-test constructed with robust standard errors clustered at the subject level. Multivariate analysis explaining the waste amount is performed via a censored (tobit) regression analysis, which is estimated via maximum likelihood and features standard errors clustered at the subject level. A censored regression analysis is chosen because the dependent variable features a high percentage of zeros, which is known to bias regression coefficients estimated via ordinary least squares.

**Table 2:** Summary statistics during free-living conditions.

Variables	Mean or %	SD	Min	Max
***Per Food Item***				
Plate waste (g)	5.63	37.32	0	1107
Food taken (g)	169.96	219.00	1.82	2952
Plate waste (kcal)	7.69	43.06	0	610
Food taken (kcal)	233.42	244.91	1.08	2786
% Plate waste*	2.5	11.62	0	100
% Items featuring any plate waste	5.83	0.23	0	100
% items that are a liquid	15.79	0.36	0	100
# of servings selected per item	1.86	2.35	0.2	50
Calories/serving	157.57	153.65	1	1477
Food code group (% of Items)				
1. Milk and milk products	15.17			
2. Meat, poultry, fish and mixtures	19.58			
3. Eggs	1.08			
4. Legumes, nuts, and seeds	2.17			
5. Grain products	25.58			
6. Fruits	6.33			
7. Vegetables	14.41			
8. Fats, oils, and salad dressings	4.62			
9. Sugars, sweets, and beverages	11.04			
% Calories as Fat	34.90	27.4	0	100
% Calories as Protein	14.51	14.66	0	91
% Calories as Carbs	48.61	31.48	0	100
***Per Day***				
Items selected	8.84	3.02	3.5	15.2
Calories selected	2044.53	728.24	862	3545
Calories consumed	1977.20	701.87	783	3411
Calories as plate waste	67.32	99.10	0	569
% Plate waste*	3.3			

N = 2400. Food Code groups correspond to the nine food groups in the United States Department of Agriculture’s Food and Nutrient Database for Dietary Studies (FNDDS version 3.0).

*Mean % plate waste differs between the per item and per day calculation because the per-item mean is an unweighted average across items while the per-day mean is a weighted mean across items consumed each day where item size serves as the weight factor.

### Ethics statement

This study was approved by the Pennington Biomedical Research Center (PBRC) Institutional Review Board and were registered at clinicaltrials.gov NCT 01678885. All participants signed informed consent forms after being briefed on the study and having all questions answered by research staff.

## Results

**Fig 1:**
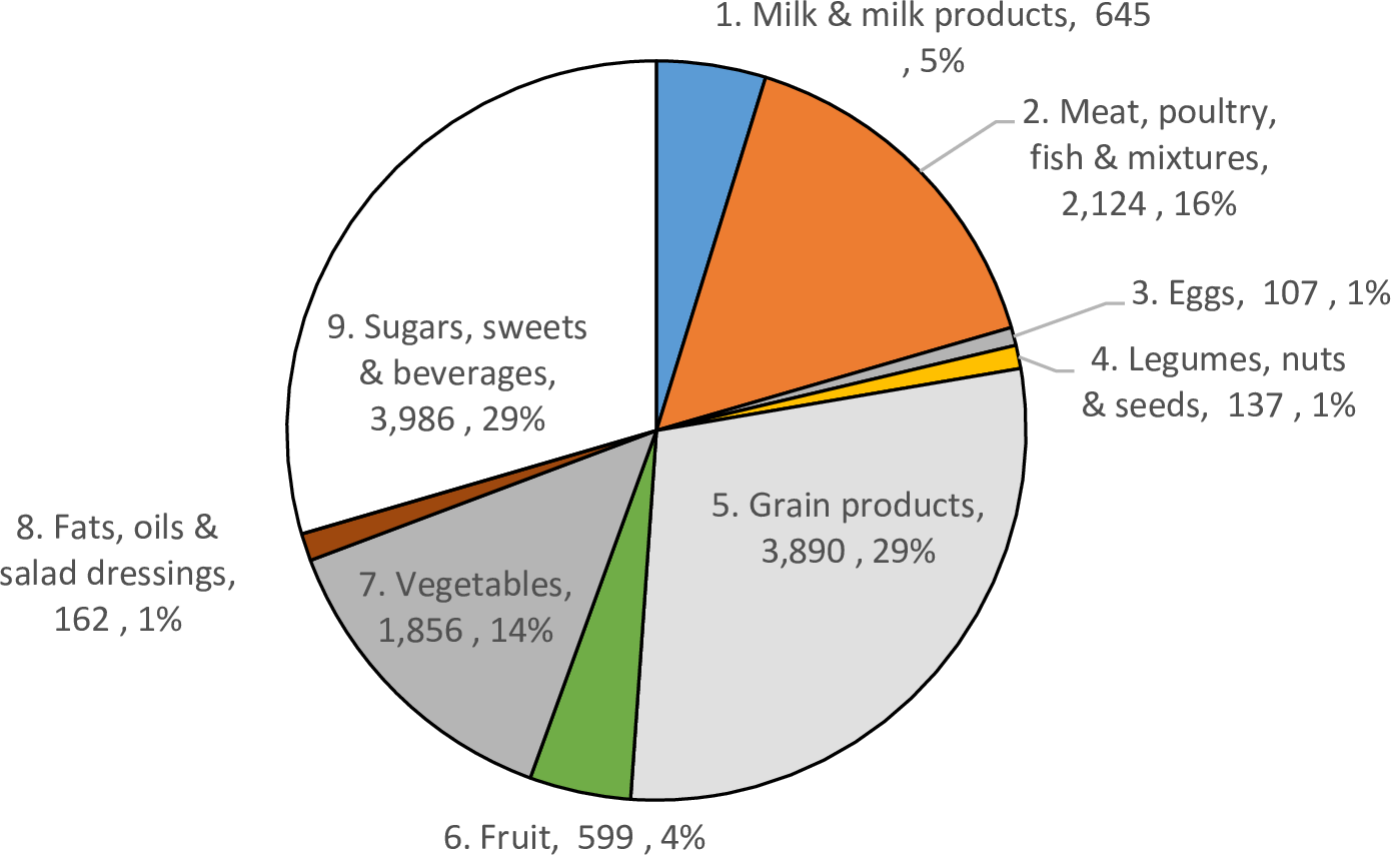
Amounts (g) and % wasted during free-living conditions.

**Fig 2:**
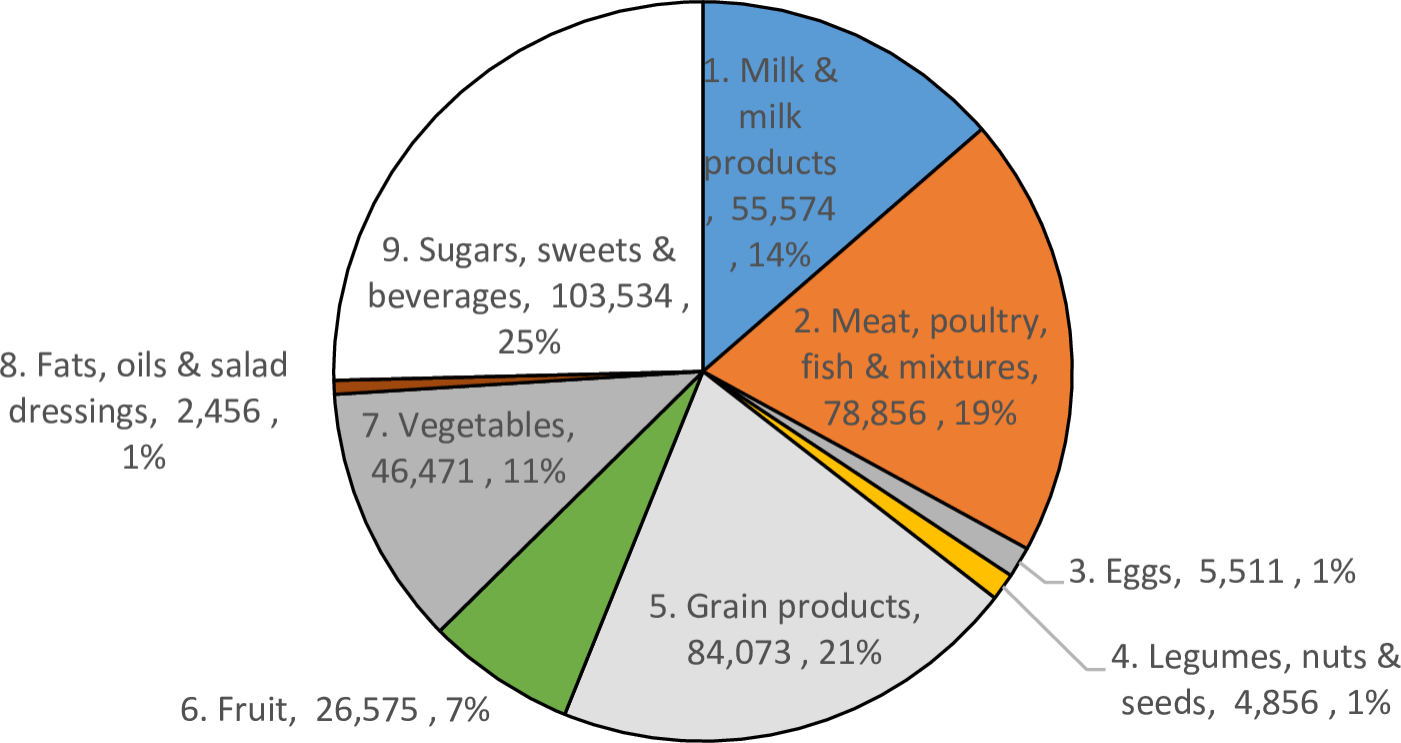
Amounts (g) and % selected during free-living conditions.

Average per-item plate waste was 5.63g (95% confidence interval 4.13g to 7.12g) and 7.69 kcals (Table 2), which constituted an average of 2.5% of the amount served. 6% of all items served generated plate waste. Of the items served 16% were classified as liquids while 26% were grain products, 20% were meat, poultry, fish and mixtures, 15% milk and milk products, 14% vegetables, 11% sugars, sweets, and beverages and 6% fruits. Figs 1 and 2 display total waste and selection by food category. The greatest waste amounts across the sample are classified as sugars, sweets and beverages (29.5%) followed by grain products (28.8%), meat, poultry, fish and mixtures (15.7%) and vegetables (13.7%). The largest constituent of waste attributable to sugars, sweets and beverages is waste from sugar-sweetened drinks, which constitutes 82% of the waste in this food group in this sample.

Across the 2,400 items of food selected by the 50 participants during the course of the study, the average item consisted of 1.86 servings (233 kcals and 170g, Table 2). For the average item, the calories were constituted as 14.5% protein, 34.9% fat, and 48.6% carbohydrate. An average of 8.8 items were selected each day for an average daily selection of 2,044.5 kcal with 67.3 kcal or 3.3% of selected energy resulting in plate waste and average daily intake of 1,977.2 kcal. Note that the 2.5% plate waste figure from the per-item statistics is smaller than the 3.3% daily figure because the different implicit weighting approaches to calculating the two figures. Specifically, the per-item figure is an average kcals across items where larger and smaller items receive equal weighting whereas 3.3% is a daily figure in which items containing more total calories are implicitly weighted more heavily in the calculation.

Pairwise comparisons of the amount of plate waste by demographic groups is presented in Table 3 as are correlation coefficients for the continuous variables of age and BMI. No differences or correlations are significant at the 5% level. Pairwise comparisons by food groups versus items outside of the listed food group are presented in Table 4 (top panel). Milk and milk products featured lower amounts of plate waste than other items.

**Table 3:** Per-Item plate waste by subject demographics.

	N	Plate waste g	*p*
*Category*			
Male	343	4.02 (1.37)	0.467
Female	2057	5.90 (0.86)	
< 50 years	1407	5.25 (1.07)	0.616
≥ 50 years	993	6.16 (1.04)	
BMI < 30 kg/m^2^	827	5.00 (0.99)	0.955
BMI ≥ 30 kg/m^2^	1573	5.95 (1.04)	
White	1547	4.53 (0.93)	0.183
Non-white	846	7.61 (1.33)	
*Pairwise Correlation Coefficients*			
Age (years)		0.0001	0.776
BMI (kg/m^2^)		0.016	0.779

Top panel features category means (standard errors). *p*-values correspond to *t-*tests between category means based on standard errors clustered at the participant level. Bottom panel features pairwise correlation coefficients between the continuous variables and the plate waste measures where the *p*-values are based on standard errors clustered at the participant level against the null of zero correlation.

**Table 4:** Plate waste by item type and content.

Group	N	Mean Plate Waste (g)	*p*
Liquids	379	12.67 (3.93)	0.741
*Food Code Groups*			
1. Milk and milk products	364	1.77 (0.86)	**0.004**
2. Meat, poultry, fish and mixtures	470	4.52 (1.00)	0.615
3. Eggs	26	4.12 (4.12)	0.676
4. Legumes, nuts, and seeds	52	2.64 (1.59)	0.826
5. Grain products	614	6.34 (1.30)	0.098
6. Fruits	152	3.94 (2.59)	0.135
7. Vegetables	346	5.36 (1.27)	0.470
8. Fats, oils, and salad dressings	111	1.46 (0.39)	0.805
9. Sugars, sweets, and beverages	265	15.04 (5.33)	0.345
Servings Selected > 1	944	9.89 (1.80)	**0.000**
Cal/serving > 119	1194	8.66 (1.39)	**0.001**
% Cal as Protein > 35%	1200	5.17 (0.79)	0.578
% Cal as Fat > 9%	1207	3.79 (0.60)	0.411
% Cal as Carb > 48%	1200	7.25 (1.39)	0.300
*Correlation Coefficients*			
Servings Selected		0.041	**0.039**
Grams Selected		0.220	**0.003**
Cal/serving		0.045	**0.001**
% Cal as Protein		-0.019	0.671
% Cal as Fat		-0.064	0.230
% Cal as Carb		0.068	0.311

Top panel features means (standard errors) in columns 3. Liquids include items from several of the food code categories, including milk, fruits and beverages. *p*-values in column 4 are from *t-*tests that use standard errors clustered at the subject level and are robust to non-normal distributions. Bottom panel features pairwise correlation coefficients between the continuous variables and the plate waste measures where the *p*-values are based on standard errors clustered at the participant level against the null of zero correlation. *p-*values ≤ 0.05 are bolded.

Items where more than one serving is selected yield greater amounts of plate waste than do items where a single serving or less is selected. Items with caloric density above the sample median (> 119 kcal/serving, e.g., beef steak, pizza, lamb chop) are wasted in larger amounts than other items. Correlational analyses between plate waste and the continuous variables (bottom panel, Table 4) largely confirms the insights drawn from the group analysis in the top panel and reveals a significant correlation between amount of plate waste and grams selected.

Multivariate analysis (Table 5) confirms several pairwise insights, including that plate waste is significantly positively associated with the number of grams selected, and that milk and milk products (the omitted food group in the regression) are associated with significantly lower waste amounts than for vegetables; fats, oils, and salad dressings; and grain products.

**Table 5:** Censored regression results: Plate waste (g) per Item in free-living conditions.

Covariate	Plate Waste (g)
Age	1.778 (0.166)
BMI	-1.321 (0.563)
Female	44.647 (0.148)
African American (White omitted)	47.132 (0.183)
Servings Taken per item	6.837 (0.070)
Grams Taken per item	**0.200** **(0.025)**
Cal/serving	0.088 (0.146)
Liquids	26.848 (0.575)
Food Code Group (group 1 omitted)	
*2*. *Meat*, *poultry*, *fish and mixtures*	98.083 (0.051)
*3*. *Eggs*	27.531 (0.804)
*4*. *Legumes*, *nuts*, *and seeds*	120.581 (0.115)
*5*. *Grain products*	**148.700** **(0.004)**
*6*. *Fruits*	52.054 (0.408)
*7*. *Vegetables*	**152.457** **(0.002)**
*8*. *Fats*, *oils*, *and salad dressings*	**143.886** **(0.028)**
*9*. *Sugars*, *sweets*, *and beverages*	87.994 (0.136)
% Cal as Protein	0.824 (0.372)
% Cal as Fat	0.235 (0.687)
Intercept	-654.826
σ	228.699
N	2400
*F*(18,2382)	1.81
McFadden Pseudo-R^2^	0.025

*p*-values based on standard errors clustered at the subject level listed in parentheses. *p–*values ≤ 0.05 are in bold. % Cal as Carb is omitted due to collinearity with % Cal as Protein and % Cal as Fat.

Table 6 features summary statistics from the laboratory meals. The meals featured an average serving of 1136 calories. Solid plate waste averaged 203g (95% confidence interval, 185.7g to 220.8g) which was 531 kcal and 39% of the amount provided. Taking the difference yields an average caloric intake of 605 calories per meal. Table 7 includes the estimates from the censored regression model, which reveals that, in the lab meal setting, women leave more plate waste than men and that the fruit salad item (the omitted item in the regression) generated significantly less plate waste than all other meal items (drink, sandwich, pretzels and cookies).

**Table 6:** Plate waste summary statistics from laboratory-based meals.

Measure	Mean	Standard Error
Amount solid plate waste per meal (g)	203.25	8.83
Amount solid plate waste per meal (kcal)	531.33	21.03
Solid plate waste (%)	39.06	1.68
Amount food served per meal (kcal)	1086.08	1.99
Amount food and liquid served per meal (kcal)	1136.40	5.53
Amount food and liquid consumed per meal (kcal)	605.07	22.19

N = 94 meals and 376 items, both taken from 50 subjects at one or two lab meals.

**Table 7:** Censored regression results: Plate waste per item in lab meals.

Covariate	Plate Waste (g)
Age	0.125 (0.766)
BMI	-0.771 (0.375)
Female	**43.335** **(0.031)**
African American (White omitted)	3.825 (0.709)
Food Item (Fruit Salad omitted)	
*Drink*	**75.324** **(0.002)**
*Sandwich*	**114.928** **(0.000)**
*Pretzels*	**28.796** **(0.005)**
*Cookies*	**22.559** **(0.029)**
Intercept	-21.354
σ	91.950
N	470
*F*(8,460)	20.03
McFadden Pseudo-R^2^	0.02

*p*-values based on standard errors clustered at the subject level listed in parentheses. *p–*values ≤ 0.05 are in bold.

## Discussion

The average level of plate waste recorded among the 50 participants in free-living conditions was 2.5% per item selected with a 95% confidence interval of 2.05% to 2.99%. Overall, 3.3% of selected food was left as plate waste. Both of these figures are lower than previously published plate waste estimates featuring adult samples (S1 Table). Qi and Roe [21] find plate waste of 11% (41g) at a free buffet provided to survey respondents; Wansink and Van Ittersum [22] estimate plate waste between 8% and 14% among paying customers at an all-you-can-eat Chinese buffet; Just and Wansink [23] estimate plate waste between 7% and 10% among customers paying half and full price at an all-you-can-eat pizza buffet; Freedman and Brochado [24] find 18% plate waste for French fries in an all-you-can-eat university dining service; and Norton and Martin [25] estimate 17% plate waste at a college dining hall. Other university plate waste studies [26–28] yield estimates of the volume of waste in the range of 63g–124g per patron per meal. Hotel breakfast buffet guests are found to generate 15g of plate waste per patron [29]. The lowest point estimate from the reported extant studies is 7%, which is more than double the figures estimated for this sample.

This difference from previous estimates may be related to several factors that differentiate our work from previously published studies. First, this study tracks plate waste of participants across all meal occasions within a day over multiple days including weekend days. Most other studies focus on a single meal, usually lunch. Previous research in the United Kingdom finds that more household food waste is generated at the evening meal than during either midday or morning meals [7].

Second, and related to the first difference, this study collects data from the same individuals over multiple meal settings and multiple days. While some of the listed studies do sample the same adult population over multiple days (e.g., [6, 7, 24–28]) or the same population under different treatments (e.g., [18, Study 3A]), individual-level data was not tracked (though [6] provides some correlational analyses of waste versus household characteristics). Hence, our ability to cluster standard errors at the individual participant level improves the precision of statistics used for inference.

Third, this study tracked both food and beverage waste, whereas much of the previous work featuring adults generally focuses on solid foods only (exceptions include [6, 7, 27]). We note that previous work focused on children in school lunch settings often tracks milk waste separately (e.g., [12, 13, 15, 16, 39]). Given that caloric liquids accounted for 10.0% of wasted calories and 35.5% of wasted grams in our sample, it suggests methods should estimate beverage as well as solid food waste to provide the most comprehensive accounting of plate waste.

Fourth, the food selected by participants in the free-living portion of this study was not exclusively obtained from buffet or institutional settings. The buffet settings in the reported extant literature allow individuals to select as much as they care to eat either for a fixed price [22–23], for free [18, 21] or as part of the rental of a hotel room [29]. The university studies occur in settings where meals are often pre-paid or all-you-can-eat [24–28], while the cost of hospital meals is likely included in overall medical expenses and item selection is likely limited due to medical and institutional constraints [40]. Furthermore, studies from household diaries [6, 7] omit meals consumed outside the house.

Another distinguishing characteristic of this study is the ability to connect plate waste data to individual characteristics. While we find no significant differences across demographic categories for items selected in free-living conditions, we do find that females leave significantly more plate waste than males in the lab meal, which is consistent with previous findings that women waste more food in dining settings (e.g., [26]). It also suggests the method holds potential for providing additional insights if applied to larger sample sizes in which more detailed individual characteristics are assessed (e.g., income, labor market status, household size, awareness and attitudes towards food, food waste, food safety, sustainability, etc.).

The RFPM® used in this study has been validated as an accurate approach for measuring energy and nutrient intake among adults in free-living conditions, and a key element of this approach is the expert assessment of photographs of plate waste. The method permits analysis on an item-by-item basis and provides details concerning both the amount selected and the amount left by participants. The 2,400 items analyzed in this study are classified into 407 distinct standard food codes, which is linked via FNDDS to provide detailed nutrient profiles for each item. Most extant studies of adults were unable to capture this degree of granularity concerning selection and waste. Exceptions include household food waste diary studies [6, 7], though food choices were not linked to detailed nutritional profiles for each item selected, and [24], though their focus was limited to a single food item.

The significant positive association between the amount of food selected and the amount of plate waste is notable. From the univariate analysis (Table 4), we find that when more than one serving is selected the amount of plate waste is 3.5 times larger (9.89 g vs. 2.86 g) and that grams of food selected is significantly positively correlated with grams of plate waste (*r* = 0.22). Both provide an indicator of the portion size selected by the subject. Larger portion sizes have been associated with greater consumption in a number of studies (e.g., [41]). However, manipulations such as larger plate sizes that stimulate larger portion size selection in self-service settings are also associated with larger levels and percentages of plate waste [22]. Also, in a study of children, we found that cafeteria specific factors, such as the provision of second servings, increased food selection and plate waste, but not food intake [19]. The current study provides another source of evidence linking portion size and plate waste, and further supports the use of the RFPM® and similar methods to: 1) quantify factors that affect food waste and/or intake, such as serving size or second servings, and 2) evaluate if policies and interventions are effectively changing food selection and other factors in an attempt to reduce food waste and/or intake.

There is significant literature on which types of food are the most likely to be found in household garbage collections with estimates of the percent of all purchases that end up in the garbage (e.g., [6]). For the case of England and Wales, the categories of food with the highest waste rates included salad (45.4%), bakery (30.7%) and fruit (26.3%). We find sugars, sweets, and beverages along with grain products to be the largest constituent of plate waste in our sample, with meats, vegetables and fruits constituting much smaller proportions. We note that estimates based on household food waste diaries [6, 7] are more comprehensive measures of food waste that include plate waste as well as other reasons for waste such as preparation waste and over-purchases of goods. Conversely, the present study used the RFPM® to provide detailed data on the plate waste of individuals, and the data collection procedures would need to be modified to capture other food waste that occurs at an individual and household level, such as waste that occurs during food preparation, refrigerator/freezer and cabinet cleanouts, discarding spoiled foods, etc. Our team is currently developing an app to assess such waste, in addition to the disposition of that waste (i.e., garbage, sink disposal, compost, etc.).

We find that the waste amounts on a per-item basis for items in the vegetable; fats, oils and salad dressings; and grain products are relatively high compared to other food groups. Plate waste studies conducted in NSLP settings often find fruit and vegetable waste rates are among the highest categories of waste, e.g., [12, 13, 15, 16, 39]. Out of the 2400 items recorded by the participants, only 108 were classified as fruits and 197 as vegetables (though many of the mixtures also contained vegetables, e.g., sandwiches with lettuce). In NSLP settings, fruits and vegetables are often a required element of the meal and students often have little choice about the fruit of vegetable served nor the amount served. Among our participants, less than 25% of the total mass of food selected was classified in the FDNNS fruit or vegetable categories, hence the result that less than 20% of wasted food and drink was from fruits and vegetable may be expected.

Finally, we note that plate waste from free-living conditions was substantially lower than plate waste observed in a lab meal among the same sample respondents. This likely reflects differences in selection flexibility, where in free-living conditions sample respondents have normal levels of autonomy in selecting food amounts while in the lab meal they were provided a relatively fixed amount of food with little flexibility in altering the amount or components. Given normal differences in caloric intake between women and men and the fixed quantities provided to all respondents, the significantly larger percent of food returned as plate waste among women is not surprising and is similar to other results in the literature [19, 26]. This study features a proportion of female participants (88%) that is higher than population averages. The reason for the higher proportion of women was due to offering weight loss treatment for overweight or obese subjects following the data collection portion of this study, and we have found that women more frequently seek weight loss treatment.

## Limitations

We do not know if the measured plate waste in this study was discarded or stored for future consumption. If a large portion was stored for future consumption and then actually consumed, it would mean that even a smaller portion of served food goes unconsumed than we report. Given this possibility, we advise interpreting the plate waste figures as an upper bound on the amount of food wasted by our sample as plate waste. Further, this study did not capture food waste that occurs during food preparation and via refrigerator/freezer and cabinet cleanouts, discarding spoiled foods, etc.

The data from this sample obtained during free-living conditions is reported at the item level and day level, but is not associated with specific eating occasions (e.g., breakfast, lunch, snack) and does not distinguish between meals taken at home versus in dine-out settings. In addition, the data is limited in that we have only the information from a single individual rather than from all individuals in a household. Adding this type of information could allow for additional insights into which dining occasions and household types yield greater waste.

The test meal provided subjects in this study contained 1136 on average, which may be more calories than most of our subjects would select in naturalistic dine-out or cafeteria settings. While such calorie levels are not unusual for some popular dine-out settings (e.g., a Big Mac, large fries and large Coke at McDonalds contains 1,340 calories [42]), it may not reflect typical waste levels the participants would normally create in dine-out settings. Finally, the sample is primarily female (88%), somewhat above national averages for BMI (30.9 vs. 28.7 for men and 31.2 vs. 29.2 for women, though Louisiana is frequently among the top five states for obesity rates), and drawn from the population of a single region, which inherently limits projections to national populations.

## Conclusions

Our study yields plate waste figures considerably lower than those in the previous published literature. Given the validity of the RFPM® for measuring food selection, plate waste, and food intake, we introduce the possibility that at least one component of total food waste (plate waste from individual eating occasions) is a smaller concern than previous thought and that a larger proportion of food waste occurs during food preparation and through discarding spoiled or unwanted food. Indeed, when our sample consumes a meal served to them in a laboratory setting with fixed quantities, the amount and percent of plate waste is substantially higher than those observed in free-living conditions and more in line with waste rates observed in National School Lunch Program settings where portion sizes and meal content are also not independently determined by the consumer. Our sample is small (n = 50), primarily female and taken from a single region, however, and future measurement is needed to establish more robustly the typical levels and patterns of plate waste generated in a variety of eating occasions and settings. Our finding that plate waste in free-living conditions increases with portion size (grams selected) reinforces results from the literature and suggests that interventions aimed at portion size control may warrant further investigation *vis a vis* possible implications for household food waste. While this study involved no manipulation concerning portion size, other studies that have manipulated plate size downward in an attempt to reduce portion size [22] found incidental reductions in plate waste accompanied the reduced portion sizes driven by the manipulation.

We also note that total (Figs 1 and 2) and per-item (Tables 4 and 5) waste amounts for milk and milk products was lower than for other categories including sugars, sweets and beverages; meat, poultry, fish and mixtures; grain products; and vegetables. Fruit also featured a modest amount of total waste for this sample. This suggests that for these categories, which are often identified as area of concern in plate waste studies for National School Lunch Program plate waste studies, the amount of plate waste is mitigated in free-living settings among adults who, in our sample, are able to choose portion sizes and exact food items. If such a pattern is validated in other studies, it may suggest that dining context and possibly age (children vs. adults) is an important driver of plate waste with lower rates for produce items when the individual has greater control over the types and amount of produce and dairy products selected at a particular dining occasion. This may suggest avenues for limiting plate waste in dining occasions away from home, i.e., perhaps encouraging restaurants and cafeterias to offer a greater variety of portion sizes and allowing more choice of individual food items in cafeterias could lower plate waste generation. Finally, we note that plate waste is but one facet of a household’s entire stream of food waste. Expanding these accurate and user-friendly methods available for food intake measurement into other aspects of household food waste warrants further exploration.

## Supporting information

S1 Table Published plate waste studies featuring adult populations. (DOCX)

S1 File (CSV)
