# Supplementary material for: Plate waste of adults in the United States measured in free-living conditions
Source: PLoS One. 2018 Feb 14;13(2):e0191813. doi: 10.1371/journal.pone.0191813 (PMC5812590; doi:10.1371/journal.pone.0191813)
Supplement: S1 Table — (DOCX) [file pone.0191813.s001.docx]

**Table S1: Published Plate Waste Studies Featuring Adult Populations**

| Study | Setting | Plate Waste (%) | Plate Waste/person/meal (g) | N | Notes |
| --- | --- | --- | --- | --- | --- |
| Wansink & van Ittersum [22, Study 2] | Chinese buffet restaurant | 7.9 | -- | 18 | Small plate condition |
|  |  | 14.4 | -- | 25 | Large plate condition |
| Williamson et al. [18, Field Study 3A] | Free lunch buffet at Executive Training Session | 8.4 | 59.5 | 40 | Permanent plate condition |
|  |  | 15.5 | 96.4 | 40 | Disposable plate condition |
| Williamson et al. [18, Field Study 3B] | Free lunch buffet at Executive Training Session | -- | 18.1 | 20 | Permanent plate condition |
|  |  | -- | 27.2 | 20 | Disposable plate condition |
| Williams & Walton [40] | Meta-analysis of plate waste studies of hospital patients | 30 | -- | -- | Median across 32 studies |
| Norton & Martin [25] | University cafeteria | 17 | -- | -- | Assessed 9% of trays returned during brunch, lunch or dinner during 10 randomly selected days |
| Freedman & Brochado [24] | University cafeteria, self-served French fries only | 18.4 |  | -- | 12 lunch periods over 4 weeks with an average of 350 students per day selecting French fries |
| Whitehair et al. [28] | University cafeteria | -- | 62.8 | 296 | Baseline data collection |
| Thiagarajah & Getty [27] | University cafeteria | -- | 124.5 | 4901 | Baseline (with trays) |
|  |  | -- | 101.5 | 4279 | Without trays |
| Kuo & Shih [26] | University cafeteria (Taiwan) | -- | 94.3 | 191 | Baseline data collection |
| Juvan et al. [29] | Hotel breakfast buffet (Slovenia) | -- | 15.2 | >15000 | Summer tourists |
| Quested & Parry [33] | Free-living conditions in the United Kingdom |  | 9.6 | 284* | Household diaries to estimate composition, includes liquids |
| Qi & Roe [21] | Free lunch buffet as part of survey study | 11 | 41 | 71 | Baseline treatment |

* This calculation also relies upon estimates reported by this group previously, including a household kitchen diary study featuring 284 respondents in [6].
